# CNN Bearing Fault Diagnosis Based on Symmetric Point Pattern Feature Fusion with Multi-Source Resonance Sparse Components

**DOI:** 10.3390/s26102995

**Published:** 2026-05-09

**Authors:** Yan Liu, Yuxuan Li, Qiang Sun, Lingrui Yang, Qitong Jia, Xiaoxun Zhu, Yan Yang, Panpan Yang

**Affiliations:** 1 Yunnan Dianneng Smart Energy Co., Ltd., Kunming 650228, China; 2 Department of Power Engineering, North China Electric Power University (Baoding), Baoding 071000, China; 3 Yunnan Power Investment Green Energy Technology Co., Ltd., Kunming 650228, China

**Keywords:** rolling bearing, deep learning, symmetric dot pattern, multi-information fusion, neural network, resonance sparse decomposition, fault diagnosis

## Abstract

To address the issue of low recognition accuracy caused by incomplete information, a CNN-based fault diagnosis method for rolling bearings using multi-source resonance sparse component feature fusion (RSSD-P) is proposed in this paper, which effectively resolves the problem of impact features being masked. In noise-contaminated environments, bearing vibration signals exhibit nonstationarity, obscuring fault characteristics. To overcome this, resonance sparse decomposition was employed to extract impact-related fault features. Furthermore, to fully utilize multi-sensor information and enhance fault representation, a symmetric dot pattern (SDP) method was introduced to fuse multi-source fault impact features, achieving effective integration of impact characteristics from multi-source vibration signals. A CNN-based approach incorporating multi-source resonance sparse component and SDP feature fusion was developed, and a bearing fault diagnosis model was established accordingly. Experimental results demonstrate that the proposed method achieves a fault recognition accuracy of 98.63% under varying operating conditions. Compared with other bearing fault diagnosis methods, the recognition precision is improved by 8.49%~17.8%, confirming its superior performance.

## 1. Introduction

Bearings are critical components in rotating machinery and are widely used in modern mechanical systems. Due to their relatively harsh operating environments, bearing failures occur frequently. In actual production, over 40% of rotating machinery failures are bearing-related [1]. Furthermore, due to the complexity of on-site noise, the impact components of fault information are easily masked by noise, thereby affecting identification accuracy. Therefore, achieving efficient and precise bearing fault diagnosis in complex environments is particularly important.

For non-stationary bearing vibration signals in strong noise environments, researchers commonly employ signal feature extraction methods to identify bearing faults. Common approaches include wavelet analysis [2], envelope analysis [3], empirical mode decomposition and its variants [4,5,6], and others. However, while these methods can extract fault information to some extent, they are often influenced by human experience and subjective factors, resulting in low diagnostic efficiency. In recent years, with the continuous advancement of intelligent mechanical diagnostic technologies, the integration of machine learning models—such as Artificial Neural Networks (ANNs), Support Vector Machines (SVMs), and Decision Trees (DTs)—with signal processing techniques has enabled intelligent identification of bearing faults. Shi et al. [7] achieved 98.5% fault diagnosis accuracy in noisy environments through wavelet denoising, principal component analysis (PCA) feature extraction, and Levy sparrow search algorithm–support vector machine (LSSA-SVM) classifier optimization. Zhang et al. [8] proposed a few-shot bearing fault diagnosis framework based on model-agnostic meta-learning (MAML). When applied to the test of new fault scenarios, the overall accuracy was up to 25% higher than that of the baseline method based on Siamese networks. However, many signal feature extraction methods struggle to effectively isolate and capture fault impact characteristics under strong field noise and multi-source interference, limiting their applicability and diagnostic effectiveness. Wang employed the Symmetrized Dot Pattern (SDP) method to visually represent bearing health status, optimizing image generation parameters to clearly depict different fault conditions. By integrating the channel attention mechanism of the Squeeze-and-Excitation (SE) network with a Convolutional Neural Network (CNN), the approach enhanced focus on key features while reducing redundant information [9]. Chen Youguang et al. [10] achieved high-precision early-stage fault diagnosis for planetary gearboxes through Multi-Energy Multi-Mode Decomposition (MEEMD) signal decomposition, SDP image feature extraction, and Deep Random Network (DRN) intelligent classification.

Recent studies have shown that SDP-CNN methods are effective for vibration-based fault diagnosis under variable operating conditions. Spirto et al. [11] enhanced gear fault features using multi-order tracking filtering before SDP-CNN, while a comparative study further showed that SDP-CNN can achieve performance comparable to time–frequency-image-based CNN methods with lower image transformation cost [12]. However, existing SDP-based studies mainly focus on single-signal or filtered-signal representations, and the collaborative fusion of multi-sensor fault impact components remains insufficiently explored.

The emergence of deep learning has effectively addressed the limitations of signal processing methods in feature extraction and the shortcomings of shallow machine learning in learning depth. Guo employed CNNs for feature extraction and analysis of rotational speed signals, proposing an improved algorithm by integrating manually selected feature values from the frequency domain. Experimental results demonstrate that this method can reliably diagnose bearing faults even under the interference of misalignment faults [13]. Wang integrated a multi-head attention mechanism with CNNs for bearing fault diagnosis. The proposed diagnostic method achieves effective bearing fault detection with relatively fewer CNN model parameters while demonstrating reasonable generalization capabilities [14]. Different convolutional kernels can be trained using CNNs to identify fault-related vibration components, but the features in raw, unprocessed bearing signals are often indistinct. Han et al. adopted a method of extracting features using wide convolution kernels and small convolution kernels respectively in the shallow layer, followed by feature fusion, and addressed the challenge of coexisting features of different scales in compound faults through a dual-layer approach, thereby effectively identifying compound faults of rolling bearings [15]. Hoang and Kang employed CNNs to convert current signals into grayscale images for feature learning. Information from the decision layer was then fused to achieve bearing fault diagnosis under steady-state conditions [16]. Furthermore, signals captured by multiple sensors provide richer information about system operation. Zhou et al. [17] proposed an intelligent bearing diagnosis method based on Short-Time Fourier Transform (STFT) and CNNs. By automatically extracting time-frequency map features and applying deep learning, they achieved a remaining life prediction accuracy of 99.45% and a fault classification accuracy of 95.83%, significantly outperforming traditional intelligent algorithms. In recent years, CNN-based multi-sensor data fusion strategies have been widely applied to comprehensively characterize equipment conditions. This approach leverages the synchronous correlation among multi-sensor data to enhance diagnostic accuracy and noise immunity [18,19]. Simultaneously, the issue of fault signature variations induced by variable loads has drawn considerable scholarly attention. Toma et al. [20] employed genetic algorithms to reduce the number of statistical features extracted from motor current signals for bearing faults. These features were then used to train and test classification methods, including k-NN, decision trees, and random forests, thereby evaluating bearing faults under variable load conditions. Konecny et al. [21] pointed out in their review that current research is generally confronted with the challenges of insufficient multi-source information fusion and the difficulty of laboratory methods in adapting to the complex on-site conditions.

From the above analysis, it can be seen that although the existing methods have promoted the development of intelligent bearing diagnosis to a certain extent, under strong noise interference and variable operating conditions, there are still problems such as the fault impact characteristics being easily overwhelmed and insufficient multi-sensor information fusion. In response to these deficiencies, this paper proposes a CNN-based bearing fault diagnosis method utilizing multi-source resonance sparse component feature fusion (RSSD-P). The innovation of this approach lies in:

(1) Multi-level resonant sparse decomposition utilizing multi-sensor signals enhances fault feature extraction.

(2) The SDP fusion method converts multi-channel time-domain signals into visual images, preserving the symmetry and global patterns of faults.

(3) CNN-based end-to-end learning significantly enhances diagnostic accuracy and generalization capabilities, offering a novel approach for multi-source heterogeneous data fusion diagnostics.

The remainder of this paper is organized as follows. Section 2 introduces the proposed bearing fault diagnosis framework based on RSSD-P feature fusion, including RSSD-based impact feature extraction, SDP image-based feature generation, multi-source feature fusion, and CNN-based fault recognition. Section 3 presents the experimental dataset, feature extraction results, diagnostic performance, and comparison with representative fault diagnosis methods. Finally, Section 4 summarizes the main conclusions of this study and discusses the limitations and future research directions.

## 2. Bearing Fault Diagnosis Method Based on RSSD-P

**Figure 1:**
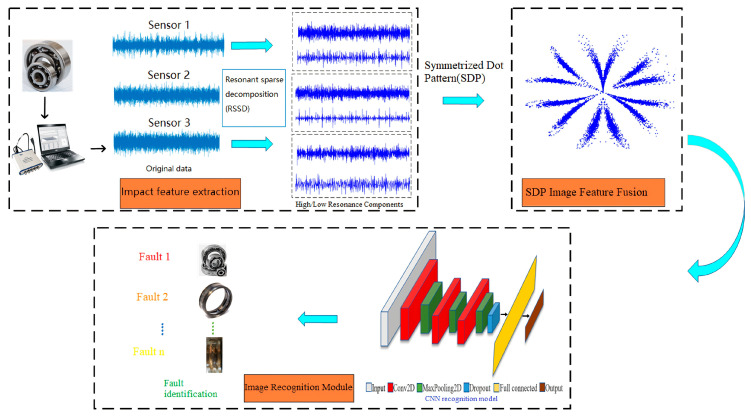
Technical roadmap of the proposed method.

The research methodology of this paper consists primarily of the following three modules:

① Impact Feature Extraction Module: Applies RSSD (Resonance Sparse Decomposition) to raw vibration data from various sensors, performs wavelet threshold denoising, and extracts high-frequency and low-frequency resonance components to reduce environmental noise interference. ② Image Fusion Feature Extraction Module: Utilizes SDP (Symmetrized Dot Pattern) to fuse high- and low-frequency resonance components from multiple sensors, generating SDP images that visually highlight differences between various faults. ③ Fusion Feature Image Recognition Module: Utilizes the SDP fusion feature image as input to construct a CNN fault recognition model, enabling the identification of bearing fault states. The overall technical framework is illustrated in Figure 1.

### 2.1. RSSD Impact Feature Extraction Module

Failure characteristics of rolling bearings typically manifest as periodic transient impacts. However, as failures worsen, substantial harmonics and sidebands emerge, making it difficult to effectively extract failure features when directly identifying the raw signal. To address this, this paper designs an impact feature separation and extraction module.

(1) Adjustable Quality Factor Wavelet Transform

Resonance sparse decomposition can separate complex signals into sustained oscillatory high-resonance components and transient-impact-containing low-resonance components based on the differing quality factors of periodic harmonic components and transient shock components within the signal [22,23].

Based on the resonance properties of the signal, high-quality factor *Q*_1_ and low-quality factor *Q*_2_, redundancy factors *γ*_1_ and *γ*_2_, and signal decomposition levels *J*_1_ and *J*_2_ are set. Using the decomposition and reconstruction filter sets of the adjustable-quality-factor wavelet transform, the signal is decomposed to generate distinct resonance property basis function libraries *S*_1_ and *S*_2_.

The formula for the quality factor *Q* is as follows:

(1) $$Q=\frac{f_{c}}{B_{w}}$$

In the formula, *f_c_* denotes the center frequency; *B_w_* denotes the frequency bandwidth.

The scaling factors *α* and *β* are given by the following formulae:

(2) $$\beta =\frac{2}{Q+1}$$

(3) $$\alpha =1-\frac{\beta }{\gamma }$$

In the formula, *γ* represents redundancy.

The formula for the maximum decomposition level *J*_max_ is as follows:

(4) $$J_{\max}=\frac{\log(\beta N/8)}{\log(1/\alpha )}$$

Finally, the signal undergoes a wavelet transform with an adjustable quality factor to obtain the initial coefficients *W*_1_ and *W*_2_ corresponding to the subbands. Combined with the wavelet basis function, these generate the initial high-resonance component *S*_1_*W*_1_ and low-resonance component *S*_2_*W*_2_.

**Figure 2:**
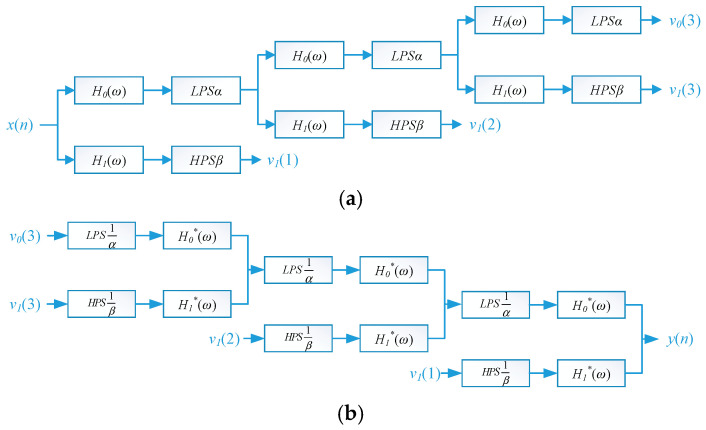
Dual-channel filter bank: (**a**) Decomposition Filter Array and (**b**) Synthetic Filter Array.

Figure 2 illustrates the decomposition and reconstruction process of signals achieved by a dual-channel filter bank using resonant sparse decomposition.

In the figure, *H*_0_(*ω*) and *H*_1_(*ω*) represent lowpass and highpass filters, respectively. *LPSα* and *HPSβ* denote low-pass and high-pass scales, respectively. *v*_0_(*n*) and *v*_1_(*n*) are subband signals with sampling frequencies *αf_s_* and *βf_s_*, respectively, where *f_s_* is the original signal sampling frequency. *α* and *β* are scale factors.

(2) High- and low-frequency resonance separation

Select appropriate weighting coefficients to establish the dissipation function formula:

(5) $$J(W_{1},\;W_{2})=\text{‖}x\text{-}S_{1}W_{1}\text{-}S_{2}W_{2}{\text{‖}}_{2}^{2}\;+\;\lambda_{1}\text{‖}W_{1}{\text{‖}}_{1}\;+\;\lambda_{2}\text{‖}W_{2}{\text{‖}}_{1}$$

In the equation, *λ*_1_ and *λ*_2_ are the regularization parameters for the two components.

After determining the regularization parameters, the optimal coefficient matrices $W_{1}^{*}$ and $W_{2}^{*}$ are obtained by iteratively solving Equation (5) using the split augmented Lagrange multiplier (SALMA) algorithm, where the dissipation function is minimized. The expressions for the high-frequency and low-frequency resonance components in the original signal are calculated using Equation (6) as *S*_1_$W_{1}^{*}$ and *S*_2_$W_{2}^{*}$, respectively.

(6) $$X=S_{1}W_{1}+S_{2}W_{2}+\mathrm{Res}$$

In the formula, *S*_1_ and *S*_2_ denote the wavelet basis function libraries for the high-resonance harmonic component and low-resonance harmonic component, respectively; *W*_1_ and *W*_2_ denote the corresponding wavelet coefficient matrices; *Res* denotes the residual component after decomposition.

### 2.2. SDP Image-Based Feature Extraction Module

**Figure 3:**
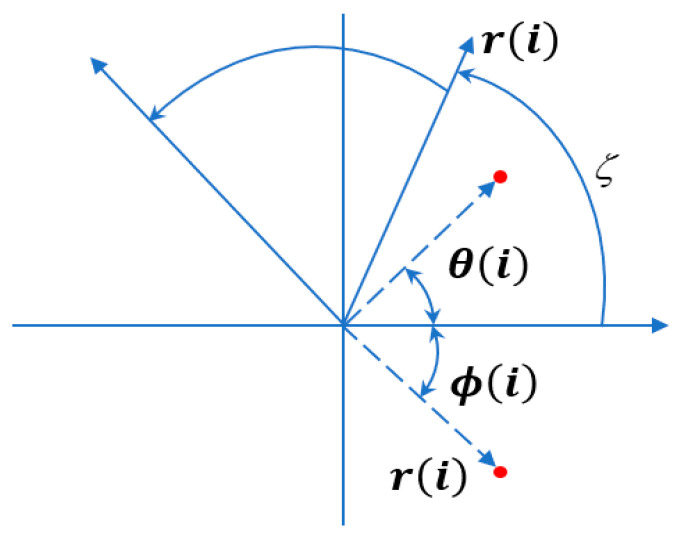
Principle of the SDP algorithm. The red dots indicate the scatter points generated by mapping adjacent time-domain signal amplitudes into polar coordinates using the SDP transformation.

The Symmetric Dot Pattern (SDP) algorithm maps complex time series as scattered points on a polar coordinate plot, enhancing the visualization of time-domain signals decomposed via RSSD through graphical representation. Its transformation principle is illustrated in Figure 3.

In the time-domain waveform, *SW_i_* and *SW_i_*_+*l*_ represent the amplitudes at time *i* and time *i* + *l*, respectively. Using the SDP method, these can be transformed into points in the polar coordinate space *S*[*r*(*i*), *θ*(*i*), *ϕ*(*i*)] [24]. In the polar coordinate space, *SW_i_* is converted into the radius component *r*(*i*) at point *i*; The adjacent point *SW_i+l_* is transformed into the angular components *θ*(*i*) and *ϕ*(*i*) of the *i*-th point. The calculation formulas for the above variables are given below.

(7) $$r(i)=\frac{SW_{i}-SW_{\min}}{SW_{\max}-SW_{\min}}$$

(8) $$\theta (i)=\theta +\frac{SW_{i+l}-SW_{\min}}{SW_{\max}-SW_{\min}}\zeta$$

(9) $$\phi (i)=\theta -\frac{SW_{i+l}-SW_{\min}}{SW_{\max}-SW_{\min}}\zeta$$

In the formula: *SW* represents the initial high-resonance component *S*_1_*W*_1_ and low-resonance component *S*_2_*W*_2_; the maximum and minimum waveform amplitudes of the original signal correspond to *SW_max_* and *SW_min_*, respectively; *r*(*i*) denotes the polar radius of the point; *θ*(*i*) and *ϕ*(*i*) denote the angles of rotation of the point around the mirror plane in the counterclockwise and clockwise directions, respectively; *θ* denotes the specified rotation angle of the mirror plane (*θ =* 360 *m*/*n*, where *m* = 1, 2, …, *n*; *n* is the number of mirror planes); and *ζ* is the gain coefficient (*ζ* < *θ*).

After SDP transformation of the decomposed time-domain signals, a “petal-shaped” scatter plot is obtained, as shown in the figure. The differences between signals are primarily manifested in: the curvature of the scatter petals, the distribution and shape characteristics of the scatter points, and the position of the geometric center.

### 2.3. RSSD-P Feature Fusion

In the SDP transformation, the mirror-plane angle *θ* determines the angular distribution of feature points, while the gain coefficient *ζ* controls the angular deviation caused by adjacent signal amplitudes. Therefore, *θ* and *ζ* should be selected to preserve the symmetry of SDP images and avoid excessive overlap or dispersion of scatter points. In this study, *θ* = 30° and *θ* = 90° are used to distinguish the high- and low-resonance components of a single sensor, respectively, and *ζ* = 20° is adopted according to the constraint *ζ* < *θ*. For multi-source fusion, six mirror planes are uniformly arranged at 30°, 90°, 150°, 210°, 270°, and 330°, corresponding to the high- and low-resonance components from the BA, DE, and FE sensors. This setting forms a symmetric six-petal RSSD-P image, which separates multi-source resonance components spatially while preserving their complementary fault information.

**Figure 4:**
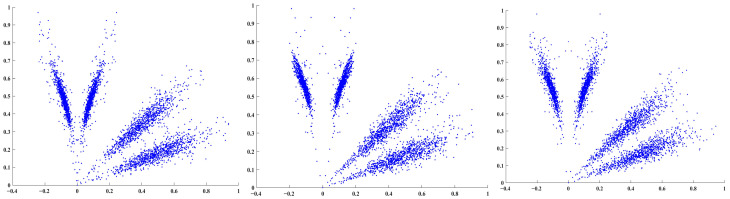
Inner race fault (BA, DE, and FE sensors).

Figure 4 shows the SDP characteristic diagram for inner ring faults (BA, DE, FE sensors). The SDP diagrams generated from high- and low-resonance components reveal distinct differences in petal opening and scatter distribution among the three fault types. The low-resonance component exhibits more pronounced petal features, demonstrating the RSSD method’s feature extraction capability. Additionally, minor variations exist among the BA, DE, and FE sensors, indicating that sensors at different positions capture slightly distinct vibration information. Analyzing signals from a single sensor often fails to comprehensively capture the equipment’s fault characteristics, which may compromise fault identification accuracy.

**Figure 5:**
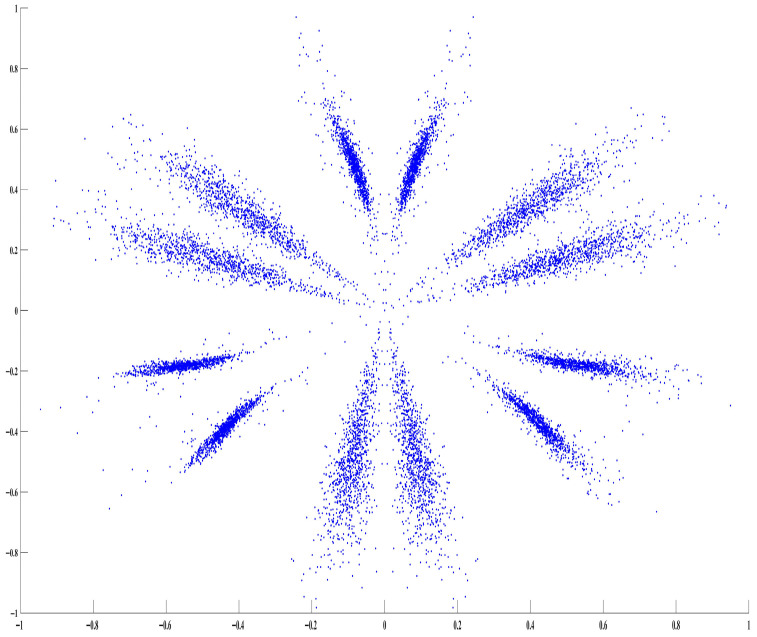
RSSD-P fusion features.

Therefore, the high- and low-resonance components from the BA, DE, and FE sensors are mapped onto the predefined six mirror planes to form the RSSD-P fusion feature image. Using the same example of three faults at the 6 o’clock position on the inner ring with a fault size of 0.1778 mm, the final RSSD-P fusion feature result is shown in Figure 5.

### 2.4. Image Recognition Module

CNN is a type of feedforward neural network widely recognized for its powerful representation learning and information extraction capabilities. To date, numerous variants have emerged. The classic CNN architecture comprises convolutional layers, pooling layers, and fully connected layers (FC).

The computation of the CNN main layer yields a mathematical representation as shown in Equation (10). Given the input *y^l^*^−1^ of layer *l* − 1, the feature map of the next layer is *y^l^*. The corresponding convolutional layer operation is as follows:

(10) yil=ReLU∑i=1Nyil−1∗kijl+bjl

Here, *N* denotes the number of convolutional kernels in layer *l* − 1, $k_{ij}^{l}$ represents the *j*th convolutional kernel in the *i*th feature map of layer *l*, $b_{j}^{l}$ denotes the bias term, and Re*LU* refers to the rectified linear unit activation function.

The pooling layer reduces the dimensionality of data extracted by the convolutional layer, thereby accelerating computation and helping prevent overfitting. This paper employs max pooling after the convolutional layer, selecting the maximum value across all pixels in the feature map.

After multiple learning iterations, image information is abstracted into higher-level image features. At the end of the network (L layer), the fully connected layer implements the final classification result. The fully connected operation is as follows:

(11) $$z^{L}=\sigma \left(w^{L}y^{L\text{-}1}+b^{L}\right)$$

Among these, *y^L−^*^1^ denotes the neuron set of layer *L* − 1, *z^L^* represents the neuron set of layer *L*, while *w^L^* and *b^L^* denote the weights and biases of the FC layer, respectively.

**Figure 6:**
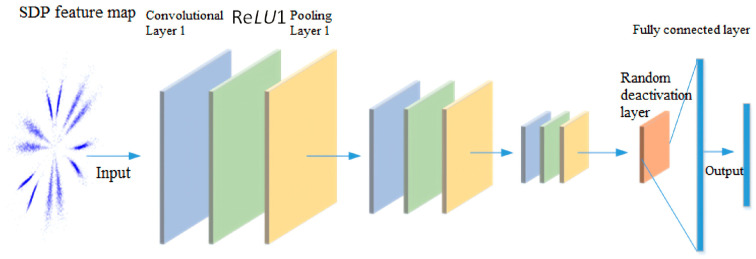
Structure of CNN.

For multi-class classification problems, this paper constructs a CNN with a relatively small number of parameters to perform the classification task. It comprises three convolutional layers, three pooling layers, and one fully connected (FC) layer. A random dropout layer is added before the FC layer to suppress overfitting. The structural diagram is shown in Figure 6.

The sizes and quantities of filters in each layer of the diagram are shown in Table 1.

## 3. Experimental Research

### 3.1. Experimental Dataset

**Figure 7:**
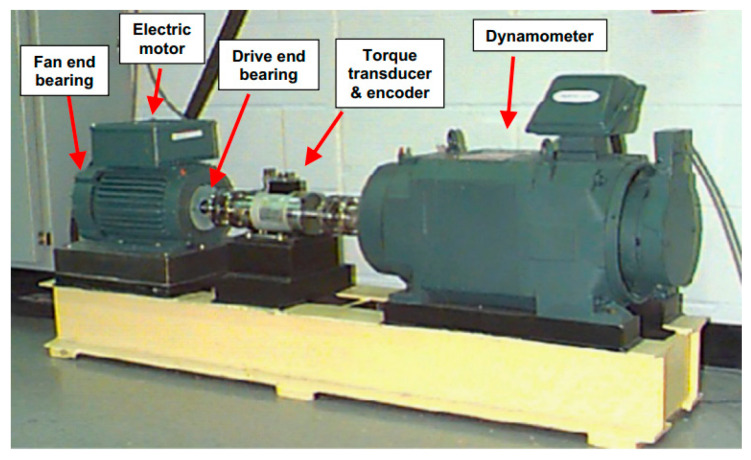
CWRU test rig.

The experimental data used in this paper are derived from the Case Western Reserve University (CWRU) rolling bearing fault vibration dataset, publicly available from the Bearing Data Center at Case Western Reserve University. The CWRU dataset is the most representative open-source dataset and has become the standard reference for validating various fault diagnosis methods in recent years [25]. The failed rolling bearing model on the CWRU experimental platform is the 6205–2 RS JEM SKF. The apparatus used for data collection is shown in Figure 7. Relevant parameters are listed in Table 2.

To simulate bearing operation under fault conditions, single-point defect flaws were manufactured via electrical discharge machining on the bearing inner ring, rolling elements, and outer ring. The defect diameters measured 0.1778 mm, 0.3556 mm, and 0.5334 mm. Experimental data collection was conducted under loads of 0 hp, 1 hp, 2 hp, and 3 hp (corresponding rotational speeds of 1797 r/min,1772 r/min,1750 r/min,1730 r/min).

This study utilizes vibration signals obtained from three accelerometers installed at the drive end (DE), fan end (FE), and base (BA) as the data source, with a sampling frequency of 12 kHz. Ten data categories are collected for each operating condition, comprising one normal data category and nine fault data categories (three fault locations × three fault magnitudes). Each category contains 500 data points, totaling 5000 vibration data entries. The dataset is randomly split into training and testing sets at a 4:1 ratio. Normal states are coded as 0, while fault locations are coded from inner to outer positions and fault magnitudes from smallest to largest, respectively assigned codes 1 through 9.

### 3.2. RSSD-P Feature Extraction from Experimental Data

**Figure 8:**
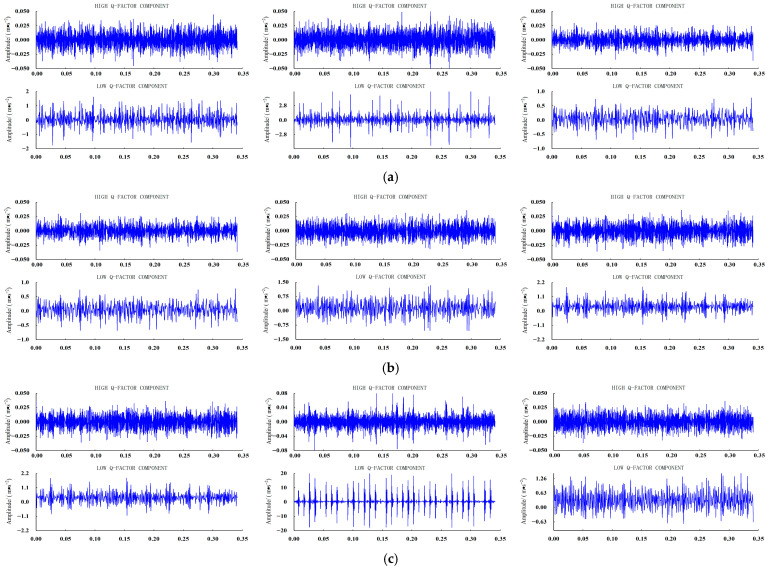
Decomposition results of RSSD for different faults: (**a**) Inner Ring Failure (Sensors BA, DE, FE); (**b**) Rolling Element Failure (Sensors BA, DE, FE); and (**c**) Outer Ring Failure (Sensors BA, DE, FE).

To demonstrate the effectiveness of RSSD, three fault data points were selected: the inner ring, rolling elements, and outer ring at the 6 o’clock position, each with a fault amplitude of 0.1778 mm. Figure 8 shows the results after applying noise reduction and Resonance Sparse Decomposition to the original signals.

It can be seen that the original signal, after noise reduction processing, successfully separates the sustained oscillatory component from the transient impact component through RSSD, making the fault characteristics more pronounced.

**Figure 9:**
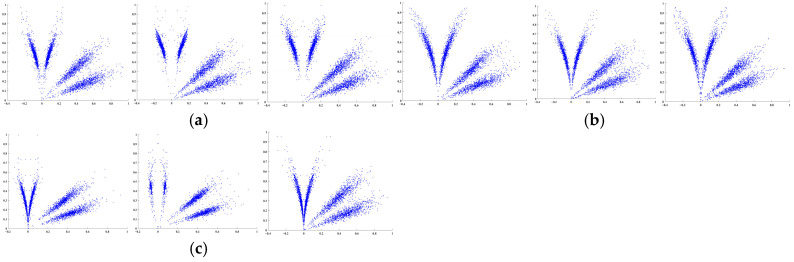
Fusion effect of SDP for high- and low-resonance components: (**a**) Inner Ring Failure (Sensors BA, DE, FE); (**b**) Rolling Element Failure (Sensors BA, DE, FE); and (**c**) Outer Ring Fault (Sensors BA, DE, and FE).

The SDP method enables the transformation of one-dimensional time series into two-dimensional images, making abstract features of one-dimensional data visualizable and intuitively revealing differences between various faults and sensor data. The corresponding high and low component SDP plots for different sensors of the three faults shown in Figure 8 are illustrated in Figure 9.

**Figure 10:**
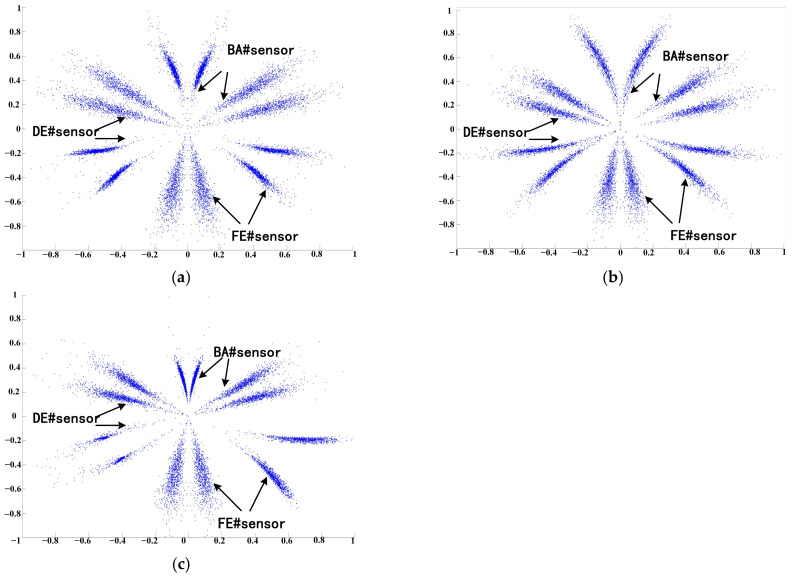
Fusion effect of RSSD-P: (**a**) Inner ring failure; (**b**) Rolling Element Failure; and (**c**) Outer ring failure.

The RSSD-P fusion features corresponding to the above high–low-resonance component fusion of SDP are as follows:

As shown in Figure 10, the SDP image fully integrates the high- and low-resonance components from the three sensors, enhancing the distinguishability of images with different fault characteristics.

### 3.3. CNN Fault Detection Model

RSSD-P images already exhibit distinct distinguishability, but manual identification of image features typically requires extensive prior learning and memorization. Moreover, subtle differences are difficult to discern, failing to meet the demands of intelligent recognition. Therefore, this paper proposes a CNN-based RSSD-P information fusion image recognition method for bearing vibration signals to achieve rapid diagnosis of rolling bearing faults. To evaluate the recognition performance of information fusion under varying operating conditions, CNN fault recognition experiments were conducted using sensor data from three positions—BA, DE, and FE—under four load conditions ranging from 0 to 3 horsepower.

**Figure 11:**
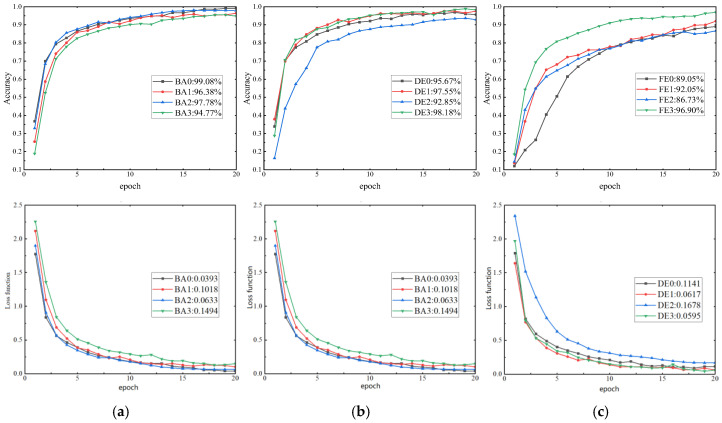
Training results of single-sensor SDP fusion for high and low components: (**a**) BA-side (0–3 hp); (**b**) DE-side (0–3 hp); and (**c**) FE-side (0–3 hp).

The final accuracy of the training process for the SDP diagram (double-petal) of high- and low-resonance components under four load conditions (0–3 hp) for a single sensor is shown in Figure 11. The overall accuracy results for the test set are presented in Table 3 and Table 4.

As shown in Figure 11, after 20 training rounds, the accuracy rates of the three sensors reached 94.77–99% for the BA end, 92.85–98.18% for the DE, and 86.73–96.9% for the FE. The training curves for BA and DE data exhibited relative stability, converging around 17 training rounds. In contrast, FE data showed greater fluctuation, with the curve indicating a slight upward trend even after 20 training rounds. Table 3 indicates that the final test set accuracy rates were 83.67–90.42% for the BA end, 86.29–92.24% for the DE end, and 73.49–85.28% for the FE end.

Combining results from the training and test sets confirms that this method possesses certain feature extraction capabilities and can provide effective learning information for CNNs. However, the accuracy of the test set shows a noticeable gap compared to the training set. Among these, the DE exhibits the highest recognition accuracy on the test set, while the FE demonstrates the lowest accuracy, increasing the risk of misdiagnosis. This discrepancy may stem from the DE sensor’s proximity to the faulty component, capturing richer vibration data, whereas the FE sensor’s greater distance yields sparser information. Consequently, without augmenting training data or employing more complex networks, integrating multi-sensor information should be considered to enhance overall data distinguishability.

**Figure 12:**
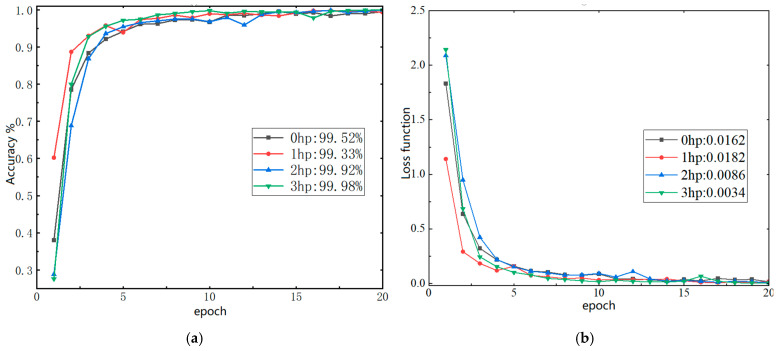
CNN training results (0–3 hp): (**a**) Accuracy rate and (**b**) Loss function.

The SDP diagram integrating high- and low-resonance component features from multiple sensors under four operating conditions. Specifically the RSSD-P diagram, shown in Figure 10, was used as input for training the CNN. The training and testing results are presented in Figure 12.

The results demonstrate that this method exhibits excellent adaptability to bearing vibration data under varying operating conditions. After 20 training cycles, the classification accuracy of the training set consistently exceeded 99%. Compared to single-sensor approaches, this method significantly enhances training accuracy while exhibiting faster convergence and greater robustness.

The test performance under varying operating conditions is shown in Table 5. The final classification accuracies under the four operating conditions reached 98.80%, 98.11%, 98.33%, and 99.21%, respectively, while the precision, recall, and F1-score remained at high levels. These results surpass the classification accuracy of a single sensor using the same method, demonstrating that the proposed approach effectively mitigates overfitting risks. Furthermore, by performing SDP information fusion on the high- and low-resonance components from multiple position sensors, the method achieves comprehensive data utilization while avoiding redundancy.

To further comprehensively evaluate the classification performance of the model under different operating conditions, the accuracy, precision, recall, and F1-score of the CNN model were calculated based on the confusion matrices under the four operating conditions, as listed in Table 5. The classification accuracies under 0 hp, 1 hp, 2 hp, and 3 hp were 98.80%, 98.11%, 98.33%, and 99.21%, respectively. The corresponding macro-averaged precision values were 98.77%, 98.14%, 98.40%, and 99.24%, the recall values were 98.74%, 98.08%, 98.38%, and 99.26%, and the F1-scores were 98.75%, 98.08%, 98.37%, and 99.24%, respectively.

**Figure 13:**
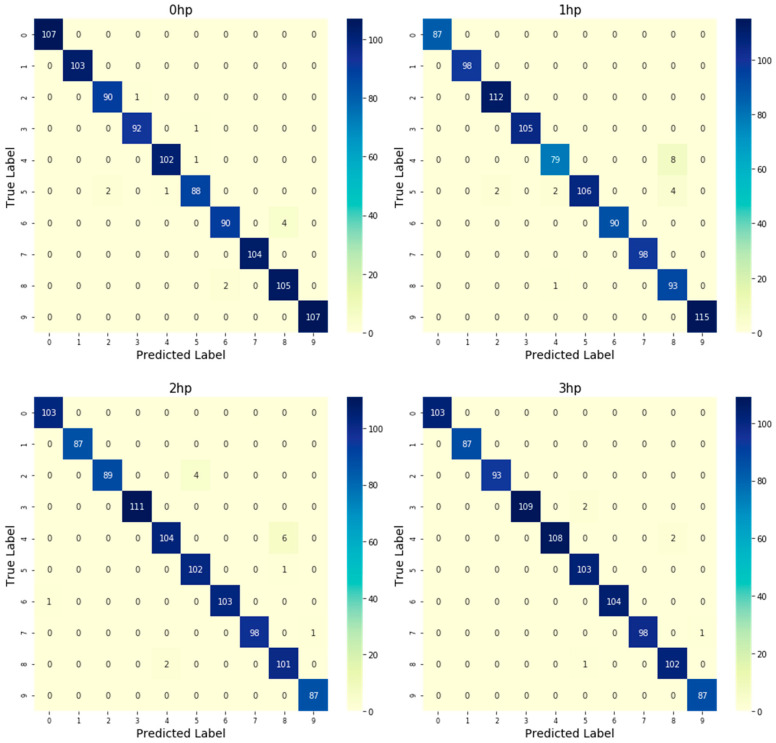
Confusion matrix analysis of CNN.

To evaluate the model’s performance more clearly and intuitively, a confusion matrix was plotted as a heatmap for the classification results of bearing failures across the four operating conditions in the test set, as shown in Figure 13.

As shown in Figure 13, most samples in the confusion matrices under the four operating conditions are concentrated on the diagonal, indicating that the model correctly identifies most bearing states. This verifies that the multi-source RSSD-P feature fusion provides highly distinguishable image representations for different fault states. Only a few samples are misclassified between adjacent fault categories, which may be attributed to the similarity of impact features under close fault locations or fault sizes. Overall, the proposed RSSD-P-CNN model maintains stable classification performance under variable load conditions, further demonstrating that multi-sensor resonance sparse component fusion and SDP image representation can effectively improve the accuracy and robustness of bearing fault diagnosis.

### 3.4. Comparison of Results

To evaluate the effectiveness of other intelligent bearing fault diagnosis methods, this section selects several common representative methods. These methods are applied to the Case Western Reserve University bearing fault vibration dataset for bearing fault diagnosis and compared with the method proposed in this paper:

(1) The EMD-SVM [26] method, which uses the EMD method for signal processing and the SVM method for state recognition;

(2) The DBN [27] method, which extracts and fuses time-domain features from vibration data collected by multiple sensors, then inputs them into a DBN network for classification;

(3) The 1D-CNN [17] method constructs a compact 1D-CNN model and uses the raw data as input;

(4) The STFT-CNN [28] method uses the Short-Time Fourier Transform (STFT) to convert vibration signals into two-dimensional time-frequency plots, which are then fed into a convolutional neural network;

(5) The RSSD-CNN method decomposes the raw vibration signal into high- and low-resonance components using RSSD, and inputs these components into a dual-stream CNN for fusion learning to obtain classification results;

(6) The RSSD-P-CNN method proposed in this paper uses the RSSD-P map—which contains fusion information derived from the high- and low-resonance components obtained via RSSD of the denoised vibration data from multiple sensors—to feed into a CNN for recognition.

**Figure 14:**
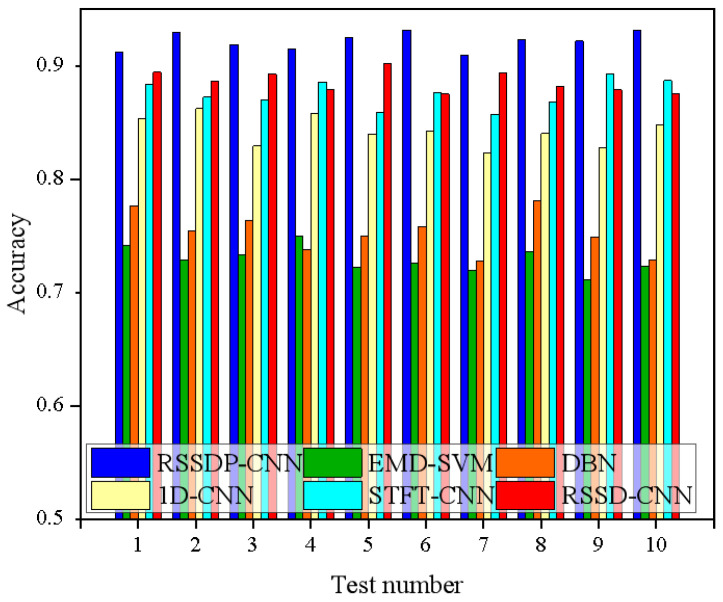
Identification Results of Bearing Fault Diagnosis Methods Under Variable Operating Conditions.

Using the average accuracy rate over ten trials as a metric, the comparison of experimental results is shown in Figure 14.

The results indicate that traditional intelligent bearing fault diagnosis methods have failed to specifically focus on the fault features concentrated in impact signals and fully extract these features, resulting in insufficient diagnostic accuracy. Furthermore, these methods fail to fully utilize the vast amount of vibration signals available in the field, resulting in a significant reduction in diagnostic accuracy under varying operating conditions. In contrast, the RSSD-P-CNN intelligent bearing fault diagnosis model developed in this paper addresses the issue of reduced accuracy under varying operating conditions by extracting vibration impact features and fusing multi-sensor impact features, thereby achieving bearing fault diagnosis with higher accuracy.

## 4. Conclusions

This study proposes a convolutional neural network (CNN)-based fault diagnosis method for rolling bearings using multi-source resonance sparse component feature fusion. First, resonance sparse signal decomposition (RSSD) is employed to separate denoised vibration signals into high- and low-resonance components, thereby enhancing transient impact features that are often masked by noise under variable operating conditions. Then, the symmetrized dot pattern (SDP) method is used to transform fault-related components from multiple sensors into two-dimensional fused images, enabling the CNN model to learn more discriminative and comprehensive fault features. Experimental results based on the CWRU bearing fault dataset demonstrate that the proposed RSSD-P-CNN method achieves high diagnostic accuracy under various load conditions, with an average recognition accuracy of 98.63%. Compared with single-sensor methods and other representative fault diagnosis approaches, the proposed method shows better robustness, improved feature separability, and stronger adaptability to variable operating conditions. These findings indicate that the integration of RSSD, multi-sensor SDP-based feature fusion, and CNN classification provides an effective strategy for rolling bearing fault diagnosis.

Despite these advantages, the proposed method still has certain limitations. First, the experimental validation is mainly conducted using a public laboratory bearing dataset, in which the fault types, operating environments, and noise conditions are relatively controlled compared with actual industrial scenarios. Therefore, the generalization capability of the proposed model under more complex operating conditions, such as variable speeds, compound faults, fluctuating loads, and severe environmental interference, requires further verification. Second, the selection of RSSD and SDP parameters can affect the quality of feature representation, and the current parameter settings are largely determined empirically. Furthermore, although the CNN model used in this study is relatively simple and efficient, its interpretability remains limited, making it difficult to fully explain the relationship between the learned image features and the physical fault mechanisms. Future work will focus on validating the proposed method using more diverse industrial datasets, developing adaptive parameter optimization strategies for RSSD and SDP feature generation, and further improving model interpretability. In addition, lightweight network architectures and online diagnostic frameworks will be explored to support real-time fault diagnosis in practical engineering applications.

## Figures and Tables

**Table 1:** Parameters of the CNN.

Hierarchy	Filter Size	Filter Size	Feature Mapping
Input	-	-	512 × 512
Convolution Layer 1	3 × 3	16	510 × 510
Pooling Layer 1	2 × 2	16	255 × 255
Convolution Layer 2	3 × 3	32	253 × 253
Pooling Layer 2	3 × 3	32	126 × 126
Convolution Layer 3	3 × 3	64	124 × 124
Pooling Layer 3	2 × 2	64	62 × 62
Dropout layer	-	-	62 × 62
Fully Connected Layer	-	128	128

**Table 2:** Specifications of 6205-2RS JEM SKF bearing.

Ball Diameter/mm	Circle Diameter /mm	Number of Rollers	Contact Angle /°	Inner/Outer Diameter /mm
25	52	9	0	25/52

**Table 3:** Test set accuracy of single-sensor RSSD-P.

Operating Conditions	Accuracy Rate/%			
	0 hp	1 hp	2 hp	3 hp
BA	90.42	88.81	83.67	88.00
DE	86.29	90.42	87.70	92.24
FE	73.79	73.49	74.50	85.28

**Table 4:** Test set loss function of single-sensor RSSD-P.

Operating Conditions	Loss Function			
	0 hp	1 hp	2 hp	3 hp
BA	0.3647	0.2852	0.6634	0.3236
DE	0.5368	0.2960	0.3532	0.3424
FE	0.7613	0.7165	0.6825	0.4744

**Table 5:** Performance of CNN on test set.

	0 hp	1 hp	2 hp	3 hp
Accuracy rate/%	98.80	98.11	98.33	99.21
Loss function	0.1516	0.0655	0.0762	0.2433
Precision rate/%	98.77	98.14	98.40	99.24
Recall rate/%	98.74	98.08	98.38	99.26
F1-score/%	98.75	98.08	98.37	99.24

## Data Availability

The data used in this study are openly available in a public repository. The original raw data were obtained from the rolling bearing fault vibration dataset of the Case Western Reserve University (CWRU) Bearing Data Center. The corresponding DOI link is: https://doi.org/10.5281/zenodo.10986655 (accessed on 6 April 2026).
